# Green Fabrication of Phosphorus-Containing Chitosan Derivatives via One-Step Protonation for Multifunctional Flame-Retardant, Anti-Dripping, and Antibacterial Coatings on Polyester Fabrics

**DOI:** 10.3390/polym17111531

**Published:** 2025-05-30

**Authors:** Zhen-Guo Zhao, Yuan-Yuan Huang, Xin-Yu Tian, Yan-Peng Ni

**Affiliations:** Qingdao Key Laboratory of Flame-Retardant Textile Materials, National Engineering Research Center for Advanced Fire-Safety Materials D & A (Shandong), Institute of Functional Textiles and Advanced Materials, College of Textiles & Clothing, Qingdao University, Qingdao 266071, Chinattxy0625@163.com (X.-Y.T.)

**Keywords:** flame retardant, chitosan derivatives, multifunctional coatings, polyester fabrics, anti-dripping

## Abstract

With the increasing urgency of petroleum resource scarcity and environmental challenges, the development of degradable bio-based flame retardants has become crucial for enhancing the fire safety of organic materials. In this work, a phosphorus-containing chitosan derivative (CS-PPOA) was synthesized via a one-step protonation reaction between chitosan (CS) and phenylphosphinic acid (PPOA) under mild conditions. The resulting multifunctional flame-retardant coating was applied to polyester (PET) fabrics. Comprehensive characterization using FT-IR, XPS, and NMR confirmed the successful protonation of chitosan amino groups through electrostatic interactions, forming a stable ionic complex. The CS-PPOA solution exhibited excellent rheological properties and film-forming ability, producing films with over 80% optical transmittance and flexibility. Thermogravimetric analysis (TGA) revealed that CS-PPOA achieved char residue yields of 76.8% and 40.2% under nitrogen and air atmospheres, respectively, significantly surpassing those of acetic acid-protonated chitosan (CS-HAc). The limiting oxygen index (LOI) of CS-PPOA increased to 48.3%, and vertical burning tests demonstrated rapid self-extinguishing behavior. When applied to PET fabrics at a 15% loading, the LOI value improved from 20.3% (untreated fabric) to 27.8%, forming a dense char layer during combustion while completely suppressing melt dripping. Additionally, the coated fabric exhibited broad-spectrum antibacterial activity, achieving a 99.99% inhibition rate against *Escherichia coli* and *Staphylococcus aureus*. This study provides a novel strategy for the green and efficient preparation of multifunctional bio-based flame-retardant coatings.

## 1. Introduction

With the intensification of petroleum resource shortages and environmental issues, the development of renewable and degradable bio-based flame retardants has become a critical direction for enhancing fire safety in organic materials [1,2,3,4]. Natural polymer materials, including chitosan [5,6,7], lignin [8,9,10], and starch [11,12,13], have garnered significant attention due to their high carbon content, multi-hydroxyl structure, and renewable attributes. These characteristics endow them with exceptional char-forming capabilities and flame-retardant potential [14,15,16]. Among these, chitosan, a primary deacetylated derivative of chitin [17], is notable for its abundant active amino and hydroxyl groups, which facilitate chemical versatility and enhance substrate interactions [18]. The high-carbon skeleton and multi-hydroxyl structure synergistically enhance carbonization efficiency. Importantly, its β-(1,4)-glycosidic bonds induce intramolecular crosslinking during pyrolysis, forming compact char layers with high residual yield [19,20]. Furthermore, its inherent cationic characteristics provide broad-spectrum antimicrobial activity [21]. By integrating renewability, environmental compatibility, and multifunctionality, chitosan offers an ideal alternative to petroleum-based flame retardants in sustainable material engineering [22].

However, the flame-retardant efficiency of standalone chitosan is inherently limited, often requiring synergistic combinations with other phosphorus or nitrogen flame-retardant systems to achieve enhanced performance [23]. In composite systems, chitosan generally functions as a carbon source and gas source to facilitate char formation. To address this limitation, researchers have concentrated on the targeted modification of its molecular structure. Specifically, the C2-NH_2_, C3-OH, and C6-OH active sites on chitosan chains offer multiple anchoring points for chemical modification [24]. By employing strategies such as acylation, esterification, and quaternization, phosphorus elements can be accurately incorporated into the molecular framework, thereby establishing an efficient multi-mechanism synergistic flame-retardant system [25]. Hu et al. [26] synthesized phosphorylated chitosan and subsequently grafted it onto the surface of nylon fabric via UV-induced graft polymerization. This was followed by the integration of the sol-gel process to develop a cross-linked coating, thereby enhancing the flame retardancy of the fabric. Li et al. [27] employed the Mannich reaction to graft phosphorous acid groups onto the amino groups of chitosan, subsequently preparing a phosphorylated chitosan (PCS)-based flame-retardant coating through urea neutralization. When applied to cotton fabric, this coating imparts rapid self-extinguishing properties. Despite the advancements in existing modification strategies, current modification technologies typically encounter challenges such as severe reaction conditions (high temperatures or extended durations), susceptibility of products to degradation, high reagent consumption, and complex purification processes for phosphorus-containing chitosan derivatives post-synthesis. These issues heighten environmental burdens. 

Moreover, in the realm of conventional chitosan phosphorylation modifications, the range of reagents employed remains relatively limited, predominantly restricted to inorganic phosphoric acid or phosphorus pentoxide. Due to the inherent flame-retardant mechanisms of these reagents, the performance of modified chitosan as a coating varies significantly across different systems. For example, it demonstrates superior flame-retardant efficiency on cellulose-based (such as cotton) fabrics but exhibits reduced effectiveness on synthetic fibers like polyester fabrics, likely due to compatibility issues between the flame-retardant mechanism and the combustion characteristics of the matrix material. Although organic phosphonate derivatives (e.g., DOPO, CEPPA) exhibit superior performance in polyester systems, conventional phosphorylation methods struggle to directly incorporate such structures. However, current chitosan phosphorylation modification approaches struggle to effectively integrate organic phosphonic acid groups. Thus, there is an urgent need for green, controllable modification processes that improve flame-retardant efficiency while optimizing environmentally friendly reactions.

Based on the principle of acid–base neutralization, the protonation reaction between organic phosphonic acids (or inorganic phosphate compounds) and chitosan’s amino groups enables the one-step synthesis of phosphorus-modified chitosan derivatives in an aqueous system. This approach eliminates the need for organic solvents or excessive acidic reagents, enabling the efficient introduction of phosphorus elements via stoichiometric reactions under mild conditions (room temperature). It exhibits excellent atom economy, avoids chitosan chain degradation, and ensures superior film-forming ability and processing stability. Additionally, the process requires no complex purification steps, significantly reducing energy consumption and costs and thereby facilitating scalable production. Moreover, the broad selection of protonating phosphonic acid agents allows for the customization of modular flame retardants by tailoring organic phosphonic acids to substrate-specific requirements and facilitates the precise optimization of compatibility and functional synergy between flame retardants and substrates.

As previously discussed, organic phosphonic acid-based flame retardants typically exhibit high efficiency in polyester textiles. Phenylphosphinic acid (PPOA), a structurally simple and easily synthesized organic phosphonic acid compound, has achieved large-scale commercial production. Its high annual output and streamlined synthesis process ensure stable supply and controllable costs. The benzene ring in PPOA enhances the thermal stability of flame-retardant coatings, while the phosphinic acid groups synergistically improve flame-retardant efficiency through gas-phase mechanisms (releasing radical quenchers) and condensed-phase mechanisms (promoting char formation). Building on this, the synthesis of chitosan derivatives via protonation with phenylphosphinic acid provides an ideal solution for practical applications, offering an economical and highly effective flame-retardant coating for polyester fabrics.

In this work, phenylphosphinic acid was selected to develop multifunctional chitosan derivatives with flame-retardant capabilities. Phenylphosphinic acid-protonated chitosan derivatives (CS-PPOA) were successfully synthesized through a one-step stoichiometric reaction between phenylphosphinic acid and chitosan amino groups in a room-temperature aqueous system. The chemical structure, solubility, rheological behavior, processability, film-forming properties, thermal stability, and flame-retardant performance of CS-PPOA were systematically characterized. Furthermore, when CS-PPOA was utilized as a multifunctional flame-retardant coating on PET fabrics, the treated fabric demonstrated exceptional flame retardancy, anti-dripping resistance, and antibacterial properties.

## 2. Experiment Section

### 2.1. Materials

Chitosan (CS, Mn = 200,000 Da) and phenylphosphinic acid (PPOA, 99%) were purchased from Aladdin Biochemical Technology Co., Ltd. (Shanghai, China). Acetic acid (analytical grade) was provided by Macklin Biochemical Co., Ltd. (Shanghai, China). All chemical reagents were used as received, without the need for further purification. The Staphylococcus aureus (*S. aureus*, CMCC(B)44102) and Escherichia coli (*E. coli*, CMCC(B)26003) strains were obtained from the China Microbial Culture Collection Center (Beijing, China).

### 2.2. Preparation of CS-PPOA

**Figure 1:**
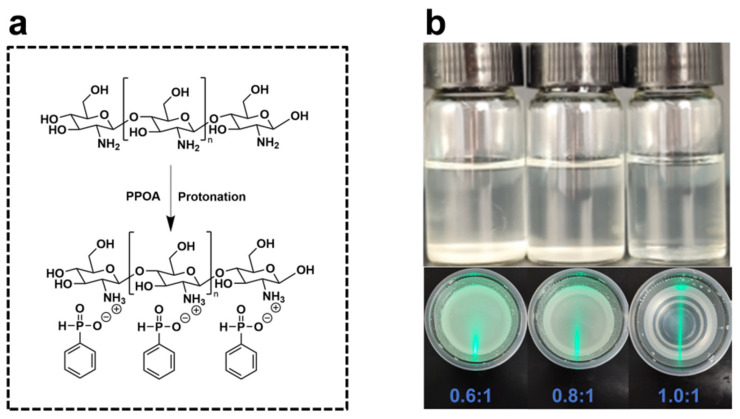
Synthetic route for CS-PPOA (**a**); solubility analysis of CS-PPOA at varying degrees of protonation (**b**).

The phenylphosphinic acid-protonated chitosan derivative (CS-PPOA) was synthesized through the protonation reaction between chitosan amino groups and phosphinic acid moieties (Figure 1). Figure 1a shows the synthesis route of CS-PPOA. The experimental procedure was conducted as follows: Chitosan and phenylphosphonic acid (with a molar ratio of amino groups to phenylphosphinic acid of 1:1) were mixed and co-dissolved in deionized water. The mixture was subjected to mechanical stirring (800 r/min) at room temperature for 3 h, during which chitosan gradually dissolved to yield a homogeneous solution, designated as CS-PPOA. For comparative studies, acetic acid-protonated chitosan derivative (denoted as CS-HAc) was prepared by dissolving chitosan in an aqueous solution containing acetic acid, maintaining identical stirring and temperature conditions.

### 2.3. Fabrication of Chitosan Derivative Films

Chitosan derivative solutions, including both CS-PPOA and CS-HAc, were cast onto clean glass plates and dried at room temperature to form free-standing films. Once the solvent had fully evaporated, the resulting films were carefully removed. Subsequently, the films were subjected to further drying and stored in a constant-temperature and humidity-controlled drying oven for subsequent applications.

### 2.4. Preparation of Flame-Retardant Coated PET Fabrics

Prior to coating, polyester fabric undergoes pre-treatment to remove surface contaminants: the polyester fabric was immersed in 50–60 °C aqueous solution containing 0.5–1.0 g/L non-ionic surfactant for 30 min under gentle agitation to strip oils and dust, followed by thorough rinsing with deionized water and drying at 80 °C to constant weight. For coating, a CS-PPOA solution with optimized rheology was then uniformly applied to the pre-treated fabric via blade-coating. After drying at 80 °C and weighing, the flame-retardant coated fabric, labeled Terylene/Px fabric (where *x* denotes the CS-PPOA loading amount), was stored in a desiccator for further testing.

### 2.5. Characterization

The rheological properties of chitosan derivative solutions were investigated through steady-state shear and dynamic oscillatory tests using an Anton Paar MCR302 advanced rheometer (Anton Paar GmbH, Graz, Austria) equipped with a parallel plate geometry system.

The particle size and zeta potential of chitosan derivative solutions were characterized using a Malvern Zetasizer Nano ZS90 (Malvern Panalytical, Great Malvern, UK).

The FT-IR spectra of the chitosan and its derivative were recorded using a Thermo Scientific Nicolet iS50 spectrometer (Thermo Fisher Scientific, Waltham, MA, USA) in attenuated total reflectance mode on solution-cast films.

The nuclear magnetic resonance (NMR) spectrum of CS-PPOA was recorded on a Bruker AVANCE III HD 400 MHz spectrometer (Bruker, Billerica, MA, USA) using *d*_6_-DMSO as the solvent.

X-ray photoelectron spectroscopy (XPS) analysis of chitosan derivatives was carried out with an Axis Supra+ X-ray photoelectron spectrometer, manufactured by Shimadzu Corporation, Kyoto, Japan.

The tensile properties were tested on an INSTRON 5967 machine (INSTRON, Norwood, MA, USA) following GB/T1040.2-2022 [28].

The optical transparency of CS-PPOA films was quantitatively measured within the wavelength range of 400–800 nm using a UV-2700 ultraviolet-visible spectrophotometer (Shimadzu Corporation).

Thermal degradation behavior was analyzed via thermogravimetry (TA Instruments TGA5500, New Castle, DE, USA) under N_2_/air atmosphere (25 mL·min^−1^ flow) with 10 °C·min^−1^ heating rate, ranging from 40 °C to 700 °C.

Flammability performance was evaluated by limiting oxygen index (LOI) testing (TTech-GBT2406-4, Testech Testing Instrument Technology Co., Ltd., Beijing, China).

The limiting oxygen index (LOI) values of CS-PPOA and coated PET fabric specimens were determined using a TTech-GBT2406-4 oxygen index tester. The samples were vertically positioned within a glass column, with the minimum oxygen concentration supporting combustion recorded through iterative nitrogen/oxygen mixture adjustments.

The vertical burning test of coated PET fabric specimens was conducted on a TTech-GBT2408 (Testech Testing Instrument Technology Co., Ltd., Beijing, China) apparatus following GB/T 5455-2014 [29] specifications. During the test, the samples were exposed to a standardized methane flame, and the after-flame time, after-glow time, and char length were meticulously recorded.

The antibacterial performance of the coated polyester fabric was assessed in accordance with the GB/T 20944.3-2008 standard [30]. In the experiment, the concentrations of *Escherichia coli* (*E. coli*) and Staphylococcus aureus (*S. aureus*) were set at 10^8^ CFU/mL and 10^7^ CFU/mL, respectively. The microbial culture medium containing the fabric samples was incubated under shaking conditions for 18 h. Subsequently, the culture medium was serially diluted by factors of 10^1^, 10^2^, 10^3^, and 104. A volume of 0.25 mL from each dilution was inoculated onto four equal portions of agar-based culture medium. The plates were then incubated at 37 °C for 24 h, after which the growth of bacterial colonies was observed and recorded.

## 3. Results and Discussion

### 3.1. Dissolution Behavior and Structural Characterization

Firstly, the influence of phenylphosphonic acid (PPOA) on the dissolution behavior of chitosan was systematically studied. As illustrated in Figure 1b, with the progressive increase in the molar ratio of PPOA to the amino groups of chitosan, the dissolution state of chitosan gradually transitioned from partial dissolution to complete dissolution. When the molar ratio reached 1:1, a stable colloidal solution with complete dissolution could be obtained. At this stage, the CS-PPOA solution system showed a significant Tyndall effect and no precipitate residue, thereby confirming the establishment of a uniform and stable dissolution system. Additionally, the pH of the CS-PPOA solution was nearly neutral, measuring approximately 6. To verify the successful preparation of CS-PPOA, multiscale spectroscopic characterization techniques were employed for comprehensive structural analysis.

**Figure 2:**
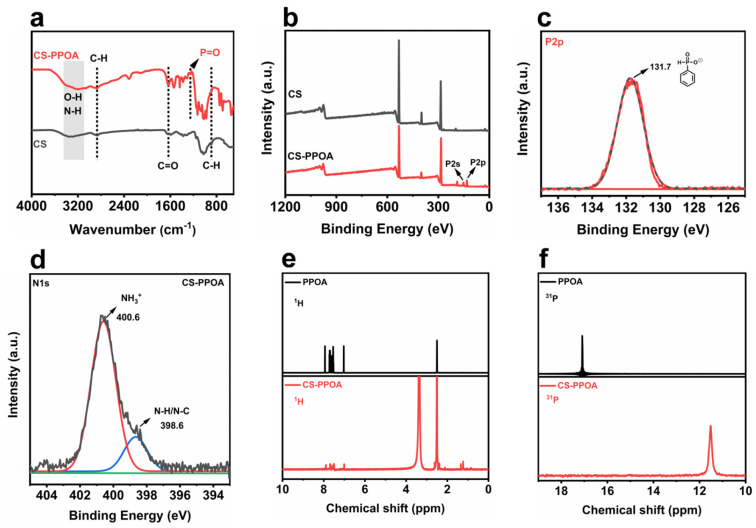
FT-IR spectra of CS-PPOA and CS-HAc (**a**), XPS survey spectra of CS and CS-PPOA (**b**), high-resolution P2p spectra of CS-PPOA (**c**), high-resolution N1s spectra of CS-PPOA (**d**), 1H NMR and 31P NMR spectra of PPOA and CS-PPOA (**e**,**f**).

A comparison of the FT-IR spectra (Figure 2a) between chitosan (CS) and CS-PPOA reveals a significant narrowing of the broad O-H/N-H coupled absorption band in the range of 3200–3600 cm^−1^. This phenomenon is attributed to the weakening or disappearance of N-H vibrational signals due to amino group protonation (-NH_3_^+^) [31,32], while the O-H vibration peaks exhibit sharper features of free and associated hydroxyl groups owing to reduced hydrogen bonding. Notably, the newly emerged P=O stretching vibration peak at 1251 cm^−1^ provides direct evidence for the successful incorporation of the phosphate group [33]. XPS analysis further revealed elemental and chemical state alterations between CS and CS-PPOA. XPS survey scans confirmed the presence of phosphorus in CS-PPOA (Figure 2b). The P2p spectrum (Figure 2c) exhibited a characteristic binding energy at 131.7 eV, corresponding to phenylphosphonate moieties. Notably, the N1s spectrum of CS-PPOA (Figure 2d) showed a dominant new peak at 400.6 eV, which is attributed to -NH_3_^+^ formation, thereby providing definitive proof of amino group protonation relative to pristine CS. Synergistic FT-IR and XPS evidence conclusively demonstrates that phenylphosphonic acid protonates chitosan’s amino groups (-NH_2_ →-NH_3_^+^) via acid–base interaction, forming an electrostatically stabilized ionic complex.

The successful synthesis of CS-PPOA was further validated by complementary ^1^H and ^31^P NMR analyses. In the ^1^H NMR spectrum (Figure 2e), all observed chemical shifts and peak integrals are in precise agreement with the target structure, thereby verifying the integrity of chitosan’s backbone following modification. Notably, the ^31^P NMR spectrum (Figure 2f), exhibits a single sharp resonance at a significantly upfield-shifted position relative to free phenylphosphonic acid (PPOA). This substantial upfield shift is attributed to enhanced electron shielding around the phosphorus atom, which results from its electrostatic interaction with the protonated amino groups (-NH_3_^+^) in chitosan. Collectively, based on the comprehensive results obtained from FT-IR, NMR, and XPS analyses, it can be conclusively stated that CS-PPOA was successfully synthesized.

### 3.2. Rheological Properties and Processability of CS-PPOA Solution

**Figure 3:**
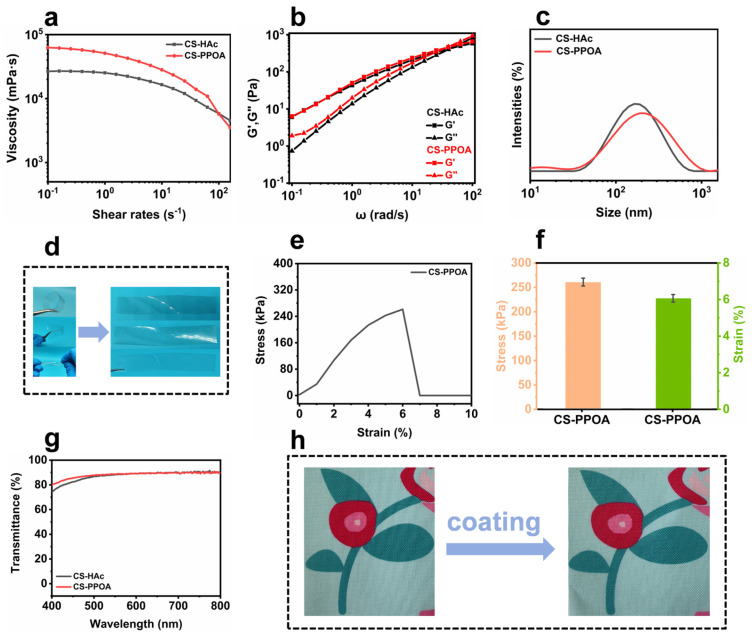
Viscosity profiles of CS-HAc versus CS-PPOA solution (**a**). Frequency-dependent viscoelastic behavior (**b**). Particle size distribution curves of CS-HAc versus CS-PPOA solution (**c**). Flexural deformation demonstration of CS-PPOA film (**d**). The tensile stress–strain curves and bar charts of CS-PPOA film (**e**,**f**). Optical transmittance of CS-HAc and CS-PPOA films (**g**). Before-and-after comparison images of the printed fabric treated with CS-PPOA coating (**h**).

The rheological properties of 5.0 wt.% CS-HAc and CS-PPOA solutions were investigated to provide guidance for optimizing its application range. The rheological properties and processing characteristics of the CS-PPOA solution are shown in Figure 3. As illustrated in Figure 3a, the steady-state shear analysis revealed that both CS-HAc and CS-PPOA solution systems exhibited typical shear-thinning behavior, characterized by a decrease in apparent viscosity with increasing shear rate. Notably, the viscosity of the CS-PPOA solution was marginally higher than that of the CS-HAc solution. This observation indicates that PPOA, akin to acetic acid, does not cause significant degradation of chitosan. In comparison with acetic acid, PPOA demonstrates a relatively lower pKa value. As a result, the ionic strength of phenylphosphonate anion is elevated, thereby promoting stronger electrostatic interactions with the protonated amino groups in chitosan derivatives. This effect contributes to an enhancement in apparent viscosity. Similarly, in oscillation rheology tests, CS-PPOA and CS-HAc solution systems showed parallel viscoelastic trends (Figure 3b), with CS-PPOA exhibiting slightly elevated storage (G′) and loss (G″) moduli. The consistency of this frequency-dependent behavior further confirmed the maintenance of chitosan’s structural integrity without excessive chain degradation. The combined rheological observations confirm that PPOA serves as an effective solvent alternative to acetic acid while potentially offering enhanced intermolecular association capabilities. To achieve a more comprehensive understanding of the solution stability and protonation behavior, the storage stability and particle size distribution of chitosan derivative solutions were systematically investigated using dynamic light scattering (DLS) and zeta potential analysis. The results are displayed in Figure 3c, and the detailed findings are comprehensively summarized in Table 1. As indicated by the particle size distribution curve, CS-HAc exhibits an average particle size of 177 nm, whereas CS-PPOA demonstrates an average particle size of 220 nm. Furthermore, zeta potential measurements provide insights into inter-particle interactions within the dispersion systems, enabling the evaluation of the stability of chitosan derivative solutions. According to the data in Table 1, both chitosan derivative solutions exhibit zeta potential values exceeding +30 mV, indicating superior dispersion stability.

Based on rheological analysis, the CS-PPOA solution demonstrates superior processability, enabling versatile fabrication into mechanically robust free-standing films via solution-casting or into lightweight aerogels through freeze-drying. The resulting CS-PPOA thin films possess exceptional mechanical flexibility, as evidenced by their remarkable resistance to damage during repeated bending and flexural deformation processes. As depicted in Figure 3d, the film retains its structural integrity after being subjected to various extreme deformations, such as 180° folding, multi-directional twisting, and high-curvature curling, with no observable cracks or delamination. Furthermore, upon the release of external stress, the film promptly restores to its original flat state via its elastic recovery mechanism. The tensile test of CS-PPOA film further demonstrated that the CS-PPOA exhibits superior film-forming properties (Figure 3e,f). Moreover, the CS-PPOA film exhibits exceptional optical transparency, achieving over 80% transmittance across the visible spectrum (400–800 nm) (Figure 3g). Its superior film-forming capability, mechanical flexibility, and high transparency make it highly advantageous for coating applications, effectively preserving the original appearance of substrates. As evidenced by the digital photograph in Figure 3h, after the application of the CS-PPOA coating, a smooth and uniform film is formed on the surface of the printed fabric, while the printed pattern remains distinctly visible.

### 3.3. Thermal Stability and Charring Performance

**Figure 4:**
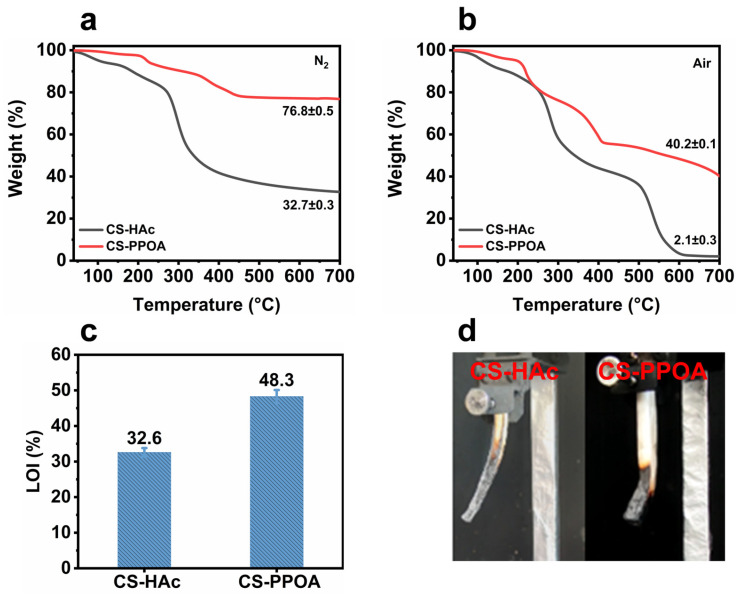
TGA curves of CS-HAc and CS-PPOA under nitrogen and air atmospheres (**a**,**b**). The LOI of CS-HAc and CS-PPOA splines (**c**). Digital photographs capturing the vertical burning behavior of CS-HAc and CS-PPOA spline materials (**d**).

The thermal stability and char-forming capacity of flame retardants serve as critical performance indicators, directly influencing their application potential and compatibility with polymer matrices. TGA analysis of CS-HAc and CS-PPOA under nitrogen/air atmospheres (as shown in Figure 4a) systematically revealed how PPOA-protonated modification enhances these key characteristics. In a nitrogen atmosphere, CS-PPOA exhibited markedly improved thermal stability. The initial decomposition temperature of CS-PPOA was 221.8 °C, which is 119 °C higher than that of CS-HAc (102.9 °C). This improvement is essential for preserving the integrity of flame retardants during material processing and application, attributable to the inherently superior thermal resistance of PPOA. Protonation of PPOA not only delays the decomposition temperature but also markedly reduces the mass loss rate at high temperatures. As a result, the char residue yield of CS-PPOA at 700 °C increases substantially to 76.8%, significantly higher than the 32.7% observed for CS-HAc. This phenomenon can be ascribed to the catalytic action of phosphonates, which promotes and intensifies the charring process of chitosan. This enhanced char-forming ability is particularly valuable for constructing protective carbon barriers during combustion [34].

The thermal decomposition behavior of CS-PPOA in an air atmosphere was further investigated. Under air (Figure 4b), CS-PPOA maintained its thermal stability advantage, with a higher initial decomposition temperature due to the robust PPOA benzene-ring structure. Notably, the thermal decomposition behavior of CS-PPOA at elevated temperatures (>250 °C) diverges distinctly from that of CS-HAc. An additional characteristic degradation peak emerges at 380.7 °C, corresponding to the breakdown of the PPOA aromatic structure, while no distinct mass-loss stage associated with chitosan backbone degradation is observed. Concurrently, CS-PPOA exhibits a significantly reduced mass loss rate. The phosphonic acid-induced dehydration carbonization effect significantly enhances the thermo-oxidative stability [35] of the resulting char layer in CS-PPOA. Even at 700 °C, CS-PPOA retains 40.2% char residue yield, which is approximately 19 times higher than that of HAc-CS (2.1%). The development of this oxygen-resistant carbon layer is critical for flame retardants functioning in real-world oxidizing environments.

### 3.4. Potential Evaluation of CS-PPOA as a Flame Retardant

Given that thermogravimetric analysis demonstrates the high charring capability of CS-PPOA, this material exhibits significant potential for application in the field of flame retardants. To systematically evaluate its performance, aerogel samples of CS-PPOA and CS-HAc were fabricated via solution casting for comparative LOI and vertical burning tests. The results are shown in Figure 4c,d. The LOI value of traditional CS-HAc samples reached 32.6%, demonstrating inherent flame-retardant properties. Remarkably, PPOA-protonated CS-PPOA achieved a substantially increased LOI of 48.3%. This improvement is primarily attributed to the optimization of thermal stability and enhanced charring efficiency through PPOA modification, facilitating the formation of compact carbonaceous barriers that effectively impede heat/oxygen transfer.

The vertical burning test further validated the modification efficacy (Figure 4d). A significant difference in burning behavior was observed between the two sample groups: the CS-HAc sample exhibited no noticeable expansion during combustion. Although it could self-extinguish after the first ignition, it continued to burn following the second ignition until it was extinguished by the fixture. In contrast, the CS-PPOA sample demonstrated outstanding self-extinguishing performance characterized by rapid intumescent char layer formation upon ignition. This dynamic charring process created porous char structures that delayed combustion propagation and enabled immediate self-extinction upon removal of the fire source [36]. The comprehensive analysis confirms the phosphorus-containing chitosan derivative CS-PPOA as a promising candidate for an independent flame retardant, with significant potential applications in the development of fire-resistant materials.

### 3.5. The Flame-Retardant Property of Terylene/Px Fabric

Based on previous research findings, the phosphorus-containing chitosan derivative CS-PPOA not only exhibits excellent flame retardancy but also demonstrates favorable rheological properties, a mild pH value, superior film-forming capability, and outstanding film flexibility and transparency [37]. These characteristics make it highly promising for applications in highly efficient flame-retardant functional coating systems. Considering the superior flame-retardant performance of phenylphosphinic acid derivatives on terylene fabrics, CS-PPOA, which contains such structures, was utilized to construct functional coatings and subsequently applied to the surface of terylene fabrics. In view of the rheological characteristics of the CS-PPOA solution, as well as the pursuit of operational simplicity and environmental sustainability, the scraping coating method was specifically adopted for the finishing process. The surface morphology of the polyester fabric was characterized using scanning electron microscopy (SEM) to compare the changes before and after the application of the chitosan derivative coating, as illustrated in Figure S1. The surface of uncoated terylene fabric fibers exhibits non-smooth characteristics, featuring numerous fine grooves and protrusions. These surface features are primarily formed during the manufacturing and processing stages of the fibers. The terylene fabric after coating exhibits markedly different surface characteristics. The coating material uniformly coats the fiber surfaces, forming a continuous film-like structure. As a result, the boundaries between fibers become less discernible, significantly enhancing the overall surface morphology of the fabric. This observation substantiates the successful fabrication of the flame-retardant coating on the fabric surface, as well as its excellent uniformity.

**Figure 5:**
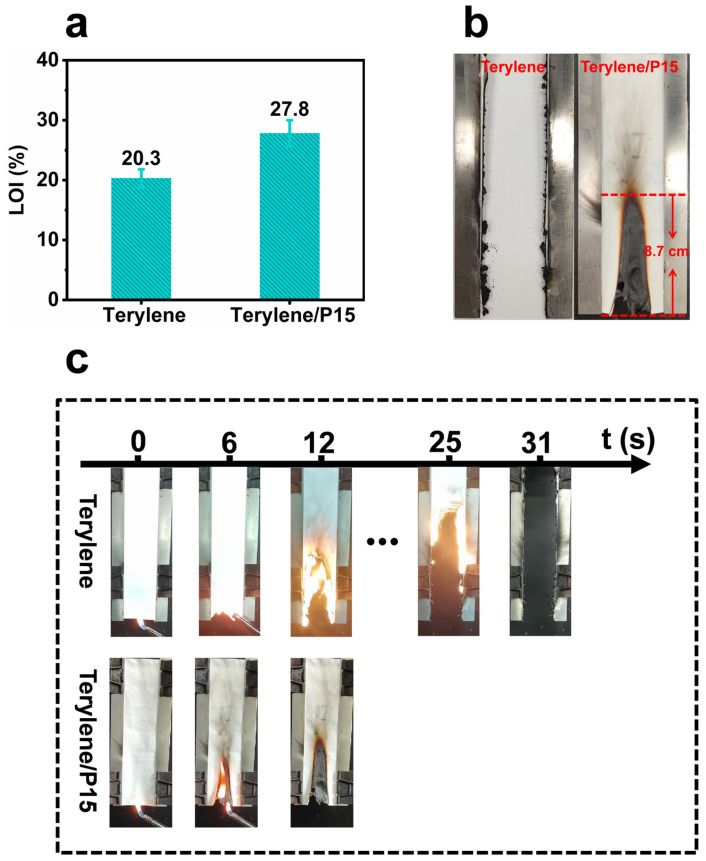
The LOI of comparison between pristine Terylene and Terylene/P15 fabrics (**a**). Post-combustion morphology of the specimens after the VFT test (**b**). Diagram of the VFT process for Terylene and Terylene/P15 fabrics (**c**).

LOI and vertical flame tests verified the outstanding flame retardancy of the PET fabric treated with CS-PPOA coating (as shown in Figure 5). At a 15% loading ratio, the LOI value of the coated fabric increased significantly from 20.3% (original terylene fabrics) to 27.8% (Figure 5a). The vertical flame test further demonstrated the significant effectiveness of the CS-PPOA flame-retardant coating in a more explicit manner. Untreated terylene fabrics ignited immediately upon exposure to flame, with rapid flame spread and complete combustion (Figure 5b,c). This resulted in almost no residue while generating substantial melting dripping. In contrast, the Terylene/P15 fabric showed slow flame propagation after ignition, accompanied by rapid formation of a dense char layer on the fabric surface that effectively suppressed flame spread [38]. The coated fabric achieved rapid self-extinguishment upon flame removal, with no after-glow or after-flame observed. The measured damaged length was only 8.7 cm, indicating the successful pass of the VFT test. Notably, the melt-dripping behavior of Terylene/P15 fabric was completely inhibited, demonstrating superior flame-retardant and anti-dripping performance. This advancement markedly enhances fire safety for PET textiles. Consequently, the CS-PPOA coating system serves as a highly effective and reliable flame-retardant and anti-dripping solution for polyester fabrics.

### 3.6. Antimicrobial Property of Terylene/P15 Fabric

**Figure 6:**
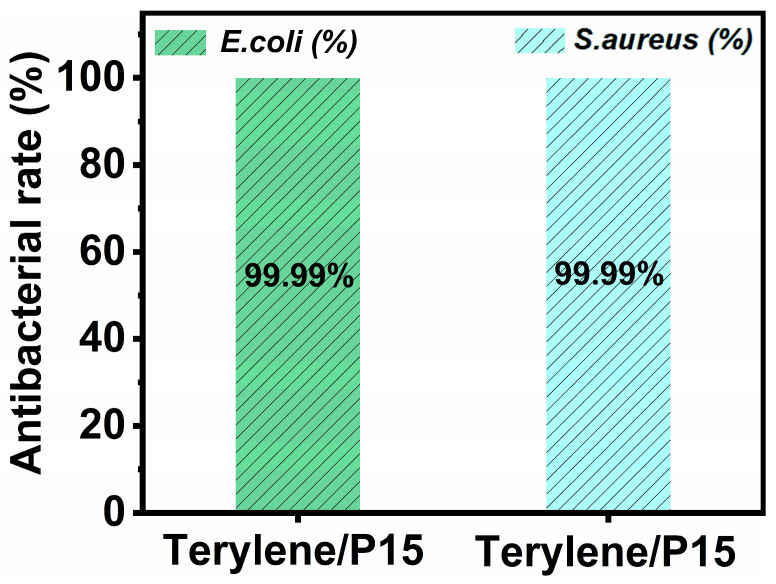
The antibacterial efficacy of Terylene/P15 against *Escherichia coli* and *Staphylococcus aureus* was evaluated.

The antibacterial performance of Terylene/P15 fabrics was systematically evaluated against representative Gram-negative (*Escherichia coli*) and Gram-positive (*Staphylococcus aureus*) strains, as shown in Figure 6. From the literature, it is evident that polyester fabric exhibits virtually no inhibitory effect on *Escherichia coli* and *Staphylococcus aureus*, with an antibacterial efficacy of 0% for both bacteria [39]. The Terylene/P15 exhibits exceptional antibacterial efficacy against both *Escherichia coli* and *Staphylococcus aureus*, achieving a bacterial inhibition rate of 99.99%. This near-complete eradication of both Gram-negative and Gram-positive pathogens highlights the broad-spectrum antimicrobial potential of CS-PPOA. The superior performance of CS-PPOA is attributed to its dual-mode synergistic bactericidal mechanism: Initially, the protonated amino groups (-NH_3_^+^) in the CS-PPOA form electrostatic interactions with negatively charged microbial cell membranes, compromising membrane integrity through structural destabilization. This primary damage enables subsequent penetration of bactericidal components into the cytoplasm, where they disrupt critical cellular processes including protein synthesis and metabolic regulation [40]. The combined effects of membrane permeabilization and intracellular interference synergistically induce rapid bacterial inactivation.

## 4. Conclusions

A novel eco-friendly and multifunctional coating, CS-PPOA, was developed by protonating chitosan with phenylphosphinic acid (PPOA). This coating integrates flame retardancy, anti-dripping, and antibacterial performance. The synthesis process is mild and highly efficient and avoids complex purification steps. PPOA modification enhances the thermal stability and char-forming efficiency of chitosan, achieving a char residue yield of 76.8% at 700 °C under nitrogen and an LOI value of 48.3%. Applied to PET fabrics, CS-PPOA formed a protective char layer during combustion, boosting the fabric’s LOI to 27.8%, enabling self-extinguishing behavior, and fully eliminating melt dripping. Additionally, protonated amino groups provide broad-spectrum antibacterial efficacy, achieving 99.99% inhibition against representative pathogens. With scalable production and multifunctional performance, CS-PPOA offers a sustainable alternative to conventional flame retardants, expanding chitosan’s application in flame retardancy and providing insights for eco-friendly coatings. This work advances chitosan-based solutions for fire safety and antibacterial applications, while future studies could focus on improving coating–substrate adhesion and extending its use to other polymer systems.

## Figures and Tables

**Table 1:** DLS test results for CS-HAc and CS-PPOA solutions.

Samples	Z-Average Size (nm)	PDI	Zeta Potential (mV)
CS-HAc	177	0.433	38
CS-PPOA	220	0.295	41

## Data Availability

The original contributions presented in this study are included in the article/Supplementary Material. Further inquiries can be directed to the corresponding authors.

## Supplementary Materials

The following supporting information can be downloaded at: https://www.mdpi.com/article/10.3390/polym17111531/s1, Figure S1: Digital photos of Terylene and Terylene/P15 coated fabrics.
