# Supplementary figures and images for: Green Fabrication of Phosphorus-Containing Chitosan Derivatives via One-Step Protonation for Multifunctional Flame-Retardant, Anti-Dripping, and Antibacterial Coatings on Polyester Fabrics
Source: Polymers (Basel). 2025 May 30;17(11):1531. doi: 10.3390/polym17111531 (PMC12158184; doi:10.3390/polym17111531)

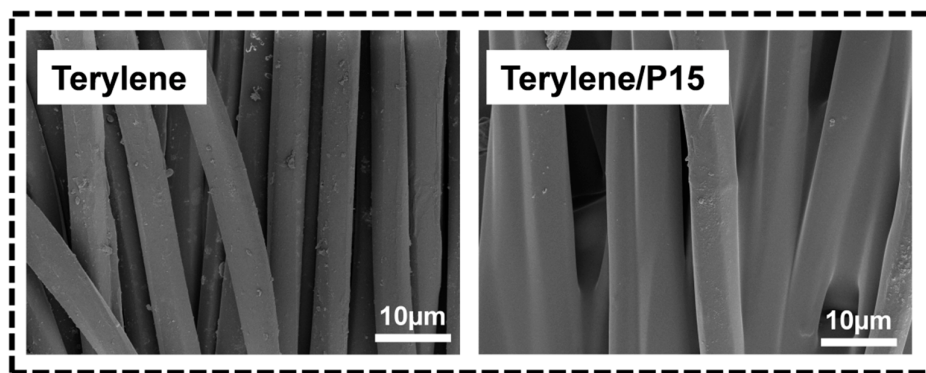

**Figure S1.** Digital photos of Terylene and Terylene/P15 coated fabrics.

Supplement: Supplementary file 1 [file polymers-17-01531-s001.zip › polymers-3665726-supplementary.pdf]
